# The Hedgehog Signal Induced Modulation of Bone Morphogenetic Protein Signaling: An Essential Signaling Relay for Urinary Tract Morphogenesis

**DOI:** 10.1371/journal.pone.0042245

**Published:** 2012-07-30

**Authors:** Ryuma Haraguchi, Daisuke Matsumaru, Naomi Nakagata, Shinichi Miyagawa, Kentaro Suzuki, Sohei Kitazawa, Gen Yamada

**Affiliations:** 1 Department of Developmental Genetics, Institute of Advanced Medicine, Wakayama Medical University, Wakayama, Japan; 2 Department of Organ formation, Institute of Molecular Embryology and Genetics, Global COE “Cell Fate Regulation Research and Education Unit”, Kumamoto University, Kumamoto, Japan; 3 Department of Molecular Pathology, Ehime University Graduate School of Medicine, Ehime, Japan; 4 Division of Reproductive Engineering, Center for Animal Resources and Development, Kumamoto University, Kumamoto, Japan; 5 Okazaki Institute for Integrative Bioscience, National Institutes of Natural Sciences, Aichi, Japan; Purdue University, United States of America

## Abstract

**Background:**

Congenital diseases of the urinary tract are frequently observed in infants. Such diseases present a number of developmental anomalies such as hydroureter and hydronephrosis. Although some genetically-modified mouse models of growth factor signaling genes reproduce urinary phenotypes, the pathogenic mechanisms remain obscure. Previous studies suggest that a portion of the cells in the external genitalia and bladder are derived from peri-cloacal mesenchymal cells that receive Hedgehog (Hh) signaling in the early developmental stages. We hypothesized that defects in such progenitor cells, which give rise to urinary tract tissues, may be a cause of such diseases.

**Methodology/Principal Findings:**

To elucidate the pathogenic mechanisms of upper urinary tract malformations, we analyzed a series of *Sonic hedgehog* (*Shh*) deficient mice. *Shh^−/−^* displayed hydroureter and hydronephrosis phenotypes and reduced expression of several developmental markers. In addition, we suggested that *Shh* modulation at an early embryonic stage is responsible for such phenotypes by analyzing the *Shh* conditional mutants. Tissue contribution assays of Hh-responsive cells revealed that peri-cloacal mesenchymal cells, which received Hh signal secreted from cloacal epithelium, could contribute to the ureteral mesenchyme. Gain- and loss-of-functional mutants for Hh signaling revealed a correlation between Hh signaling and Bone morphogenetic protein (Bmp) signaling. Finally, a conditional ablation of *Bmp receptor type IA* (*BmprIA*) gene was examined in Hh-responsive cell lineages. This system thus made it possible to analyze the primary functions of the growth factor signaling relay. The defective Hh-to-Bmp signaling relay resulted in severe urinary tract phenotypes with a decrease in the number of Hh-responsive cells.

**Conclusions/Significance:**

This study identified the essential embryonic stages for the pathogenesis of urinary tract phenotypes. These results suggested that Hh-responsive mesenchymal Bmp signaling maintains the population of peri-cloacal mesenchyme cells, which is essential for the development of the ureter and the upper urinary tract.

## Introduction

The urinary system is composed of several highly divergent organs, including the kidneys, ureters, bladder and urethra. Such organs are essential for the transfer of urine from the renal pelvis toward the bladder. Previous studies have suggested that growth factors, such as Sonic hedgehog (Shh) and Bone morphogenetic proteins (Bmps) are essential for the formation of the urinary tract [1]–[9]. The loss of such signaling in genetically-engineered mice phenocopied human urinary diseases, such as congenital anomalies of the kidney and urinary tract (CAKUT) or the VACTERL syndrome [1], [10]–[14]. The above diseases are characterized by abnormalities in the upper urinary tract. In particular, CAKUT includes a number of developmental anomalies at the level of the kidney (e.g., hydronephrosis, hypoplasia, dysplasia, duplex kidney), ureter (e.g., hydroureter), and bladder (e.g., ectopic ureteral orifice) [15]–[17]. Despite recent advances in both the prenatal diagnosis and early surgical intervention, CAKUT still remains the primary cause of kidney failure in infants (1 in 500 live births) [18]–[20].

The Shh, a Hedgehog (Hh) family ligand, controls cell fate, cell proliferation, differentiation and tissue patterning during embryogenesis [21]–[25]. The *Shh* gene is expressed in the cloacal epithelia and its signaling has vital roles for the regulation of tissue lineage within the embryonic urinary tracts such as the bladder and the external genitalia [2], [26]–[30]. The SHH protein was located in the distinct urinary tract epithelia of human embryos, in the urothelium of the nascent bladder and in the kidney medullary collecting ducts [31]. In addition, we previously suggested that the bladder mesenchyme and dorsal (upper) external genitalia are derived from the peri-cloacal mesenchyme (PCM) exposed to Hh signaling [29], [32]. However, the contribution of the PCM cells to the upper urinary tract after exposure to epithelial Hh signaling remains to be elucidated. Bmp signaling genes are also expressed in the urinary tract and are essential for urinary tract development. In fact, several mouse mutants for *Bmp* genes display various abnormalities including kidney and urinary tract anomalies [1], [33], [34]. BMP4 is known to be expressed in the nascent mesenchyme of the urogenital sinus and smooth muscle region in the human embryo [31]. Several mouse studies have suggested that biological functions of Bmp4 during urinary tract formation are considered to prevent cell death and promote the metanephric mesenchymal growth and the ureteral smooth muscle formation [1], [33], [35], [36]. The study of growth factor mediated morphogenesis requires analyses for the roles of signaling relays among different growth factor pathways. However, the spatiotemporally regulated functions of such signaling relays are unclear. In particular, it has been speculated that epithelially derived Hh signals are interpreted at the level of the surrounding mesenchyme [2], [27], [37]–[39]. Moreover, analyses of the signaling modulation in Hh signal responsive mesenchyme have not been performed.

The current study used a novel genetic analysis, the conditional ablation of *Bmp receptor type IA* (*BmprIA*) gene in Hh-responsive cell lineages. Utilizing Hh-responsive gene modulation at the level of the mesenchyme allows an analysis of the primary functions of the growth factor signaling relay. The study also revealed the cellular origin of the mesenchymal cells in the upper urinary tract by tissue contribution analyses of Hh-responsive cell lineages. Moreover, loss- and gain-of-functional analyses of Hh signaling suggested an essential correlation between Hh and Bmp signaling during urinary tract formation. In addition, the critical time-window leading to the upper urinary phenotypes including hydroureter was suggested by conditional mutation of *Shh* and *BmprIA* genes. These analyses suggest Hh-responsive mesenchymal Bmp signaling maintains the population of the PCM cells, which is essential for the development of the ureter and the upper urinary tract.

## Materials and Methods

### Mouse Strains and Embryos

The *Shh^neo^*, *Shh^flox^*, *BmprIA^flox^*, *Gli1^CreERT2^*, *Shh^CreERT2^*, *Rosa26-SmoM2* (*R26^SmoM2^*), *Rosa26R* (*R26R^LacZ^*) and *Rosa26-eYFP* (*R26R^YFP^*) alleles have been described previously [21], [23], [40]–[45]. All experimental procedures and protocols were approved by the Committees on the Animal Research at Wakayama Medical University and at Kumamoto University (*Permit Number:* 519 in Wakayama Medical University and A23-066, A23-069, A23-070 and A23-071 in Kumamoto University).

### Histological Analyses

Mouse embryos were fixed overnight in 4% paraformaldehyde (PFA/PBS), dehydrated through methanol, embedded in paraffin, and 8 µm serial sections were prepared. Hematoxylin and Eosin (HE) staining was processed by standard procedures [46]. X-gal staining was carried out as described previously [29], [47]. X-gal stained samples were embedded in paraffin and sectioned. Immunohistochemical analyses were performed by standard procedures using the antibodies (Ab): anti-GFP Ab (1∶400, Abcam), anti-GFP Ab (1∶100, Roche), anti-smooth muscle myosin (SMM) Ab (1∶200, Biomedical Technology), anti-phosphorylated-Smad1/5/8 (pSmad) Ab (1∶300, Cell Signaling), anti-Pax2 Ab (1∶200, Zymed), anti-Uroplakin3 (UPIII) Ab (1∶200, Progen), anti-alpha smooth muscle actin (SMA) Ab (1∶400, Sigma). Signal amplification for pSmad staining was performed using the appropriate ABC kit (Vector Laboratories) and the immunocomplexes were detected with DAB staining. Staining for GFP, SMM, Pax2, UPIII and SMA was visualized using Alexa Fluor 488 or 546 IgG against the primary antibodies (1∶300, Molecular Probes). Nuclear counterstaining was performed using Hoechst33342 (Sigma).

### *In situ* Hybridization for Gene Expression Analyses

Whole-mount and section *in situ* hybridizations for gene expression analyses were performed as previously described [26], [46]. The antisense riboprobe templates have been described previously: *Shh* (kindly provided by C. Shukunami, Kyoto University, Japan), *Gli1*, [48], *Pax2* [49], *Tbx18* (kindly provided by T. Suzuki, Nagoya University, Japan).

### Tamoxifen Administration for Conditional Gene Recombination

Noon of the day of a vaginal plug was designated as E0.5. A 20 mg/ml stock solution of tamoxifen (Sigma) was prepared in corn oil [29]. The pregnant females received 2 mg of tamoxifen per 40 g maternal body weight using a gavage needle at the indicated time points.

### Genetic Tissue Lineage Analysis Utilizing the Rosa26 Reporter Mouse Strains

Tissue lineage analyses were conducted by utilizing *Gli1^CreERT2/+^; R26R^LacZ/+^* and *Gli1^CreERT2/+^; R26R^YFP/+^* mice. The *Gli1^CreERT2/+^* male mice were crossed with *R26R^LacZ/LacZ^* or *R26R^YFP/YFP^* Cre indicator female mice [42], [44], [45]. Mouse embryos were processed for X-gal or immunohistochemical staining.

## Results

### Requirement for Sonic Hedgehog Signaling during Urinary Tract Formation

**Figure 1:**
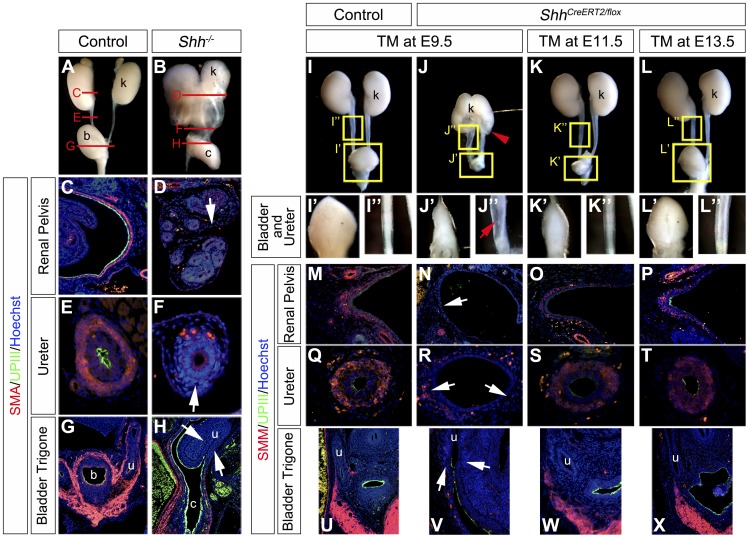
Urinary tract malformation and reduction of marker expression in *Sonic hedgehog* (*Shh*) mutant embryos. Gross morphology of the urinary tracts in the control (**A**) and *Shh^−/−^* (**B**) embryos at E18.5. Red lines in **A** and **B** indicate positions of transverse sections in **C–H**. Immunohistochemistry for the expression of alpha smooth muscle actin (SMA: red) and Uroplakin3 (UPIII: green) in transverse sections of the renal pelvis (**C, D**), the ureter (**E, F**) and the bladder trigone regions (**G, H**) at E18.5. White arrows in **D, F** and **H** indicate reduced expressions of these markers. Gross morphology of the urinary tracts of *Shh^CreERT2/flox^* embryos at E16.5, which were treated with TM at E9.5 (**J, J’, J”**), E11.5 (**K, K’, K”**) or E13.5 (**L, L’, L”**). The control embryo was treated with TM at E9.5 (**I, I’, I”**). **I’–L’** and **I”–L”** indicate higher magnification views of yellow boxes in **I–L**. E9.5-TM treated embryos displayed severe phenotypes in the kidney, the bladder and the ureter (**J, J’, J”**). A red arrowhead in **J** indicates a hydronephrosis phenotype. A red arrow in **J”** indicates the hydroureter phenotype. Immunohistochemistry for the expression of smooth muscle myosin (SMM: red) and UPIII (green) in transverse sections of the renal pelvis (**M–P**), the ureter (**Q–T**) and the bladder trigone region (**U–X**). White arrows indicate reduced expressions of differentiation markers in mutants with E9.5-TM treatment. b: bladder, c: cloaca, k: kidney, u: ureter.

Mouse mutants for the *Sonic hedgehog* (*Shh*) gene display several abnormalities in the urogenital organs [2], [26], [28]–[30]. The urinary specimens of *Shh* conventional mutants (*Shh^−/−^*) displayed prominent hydroureter and hydronephrosis phenotypes (Fig. 1A,B). *Shh^−/−^* embryos also displayed a fusion of the bilateral kidneys at low frequency (Fig. 1B) [2]. This horseshoe-like kidney phenotype is considered to be caused by Shh derived from the notochord [50]. In addition, prominent ureter dilation and bladder hypoplasia were observed (Fig. 1B; data not shown). The mutant ureters were not only dilated but also displayed a severely reduced length longitudinally between the kidney and the bladder (Fig. 1B). We next performed expression analyses for several tissue differentiation markers. The ureter, renal pelvis and bladder regions develop several tissues such as transitional epithelia, connective tissues and smooth muscles. Smooth muscles of the ureter have been suggested as important for regulating proper peristaltic movement for the urine transport. One of the causes for hydronephrosis and hydroureter is considered to be derived from abnormal smooth muscle formation of the urinary outflow tract in the ureters, renal pelvis and bladder trigone [51]. The expression of alpha smooth muscle actin (SMA: red), one of the smooth muscle cell differentiation markers, was investigated by immunohistochemical analyses (Fig. 1C–H). Its prominent expression was detected in the ureter and in the renal pelvis mesenchyme of the control embryos at E18.5 (Fig. 1C,E). In contrast, a small number of SMA positive cells were observed as dotted signals in the mesenchymal layer around the urothelium of *Shh^−/−^* embryos at E18.5 (Fig. 1D,F; white arrow). The expression in the bladder wall was also reduced in such mutants (Fig. 1G,H). These samples were co-stained with SMA (red) and Uroplakin3 (UPIII: green), which is a family of transmembrane proteins selectively expressed in the ureter epithelium and required for its water-impermeable properties [52], [53]. A reduced UPIII expression was observed in the renal pelvis and the ureter of *Shh^−/−^* embryos (Fig. 1C–F). However, the expression of UPIII in the bladder urothelium was similarly detected in *Shh^−/−^* embryos (Fig. 1G,H).

We also analyzed the expression patterns of several Hh signaling genes (Fig. S1). The *Shh* was expressed prominently in the cloaca and the bladder epithelium in E10.5-E13.5 embryos (Fig. S1B,F,J). A faint *Shh* expression was detected around the early kidney and the ureter from E11.5 onward (Fig. S1D,E,H,I; red arrowheads) [2]. The prominent expression of *Gli1* gene, a readout for Hh signaling, was observed in most of the mesenchymal layers adjacent to the *Shh*-positive cloacal epithelia and the presumptive ureters (Fig. S1K–T).

The upper urinary tract phenotypes of *Shh^−/−^* embryos were observed from E12.5 onward. We next assessed the expression of *Pax2* gene, a developmental gene in the urinary tracts. *Pax2* is expressed both in the ureteric bud and the condensed mesenchyme surrounding it in the metanephros at E11.5 [54], [55]. The expression of *Pax2* was detectable in both the control and *Shh* mutant urinary tracts (Fig. S2A–H). Its expression was not significantly altered in the metanephric mesenchyme, ureteric bud epithelium, ureter and early kidney region of *Shh^−/−^* embryos at E10.5, E11.5 and E12.5 (Fig. S2B,D,F,H). We also analyzed the expression of *Tbx18* gene, which is expressed in the smooth muscle progenitor cells of the urinary tract and known as regulating their differentiation [56]–[58]. *Tbx18* expression was decreased in the mesenchyme around the nephric duct of *Shh^−/−^* embryos in comparison to the control at E10.5 and E11.5 (Fig. S2I–L; red arrows). The significantly reduced *Tbx18* expression was observed in the mutant mesenchyme around the ureter and the early kidney at E12.5 (Fig. S2N,P; red arrows).

### The Conditional Inactivation of *Shh* Gene Led to Malformations in the Upper Urinary Tract

The prominent urinary phenotypes of *Shh^−/−^* embryos suggest the essential functions of Shh signaling for the upper urinary tract formation (Fig. 1A–H). To examine the temporal necessity of Hh signaling for the urinary tract formation, we analyzed the urinary tract phenotypes of *Shh* conditional mutants. These experiments utilized *Shh flox* (*Shh^flox^*) allele and *Shh^CreERT2^* allele, which is a tamoxifen (TM) inducible form of Cre recombinase expressing strain [23], [41], [59]–[61]. Pregnant female mice were treated orally with TM. The *Shh^CreERT2/flox^* embryos exhibited upper urinary tract hypoplasia, kidney and ureter defects at E16.5. The urinary tracts of *Shh^CreERT2/flox^* embryos reproduced the milder phenotypes of *Shh^−/−^* embryos by E9.5-TM treatment (Fig. 1J–J”). We further examined *Shh^CreERT2/flox^* embryos with TM treatment at E11.5 and E13.5 (Fig. 1K–L”). These *Shh^CreERT2/flox^* embryos exhibited slight hypoplasia of the urinary tracts (Fig. 1K–L”). The cell differentiation status of these *Shh^CreERT2/flox^* embryos was examined by smooth muscle myosin (SMM) and UPIII expression. The SMM expression was reduced in the renal pelvis, ureter and bladder trigone region in E9.5-TM treated mutants (Fig. 1N,R,V; white arrows). A small number of SMM positive cells were observed in the bladder wall (Fig. 1V). Such mutants also showed reduced UPIII expression in the dilated renal pelvis and in the ureter (Fig. 1N,R). However, its expression in the bladder urothelium was not altered in the mutants (Fig. 1V). Mutants with later-staged TM treatment, such as E13.5-TM treated mutants, showed weak expression of these markers in the renal pelvis, ureter and bladder wall (Fig. 1O,P,S,T,W,X). These results suggested that urinary tract phenotypes are caused by defective Shh signaling at early embryonic stages. Moreover, we confirmed the reduced Shh signaling in E9.5-TM treated mutants by *Gli1* expression analyses (Fig. S3). Its expression in mutants was decreased in the ventral bladder mesenchyme (Fig. S3B,D; red arrowheads), but not in the bladder trigone region and the ureter at E14.5 (Fig. S3D,F). These results suggested that ureteral *Shh* was not affected in mutants with E9.5-TM treatment.

### Hedgehog Signal Responsive Cells can Contribute to the Upper Urinary Tract from the Early Peri-cloacal Mesenchyme

**Figure 2:**
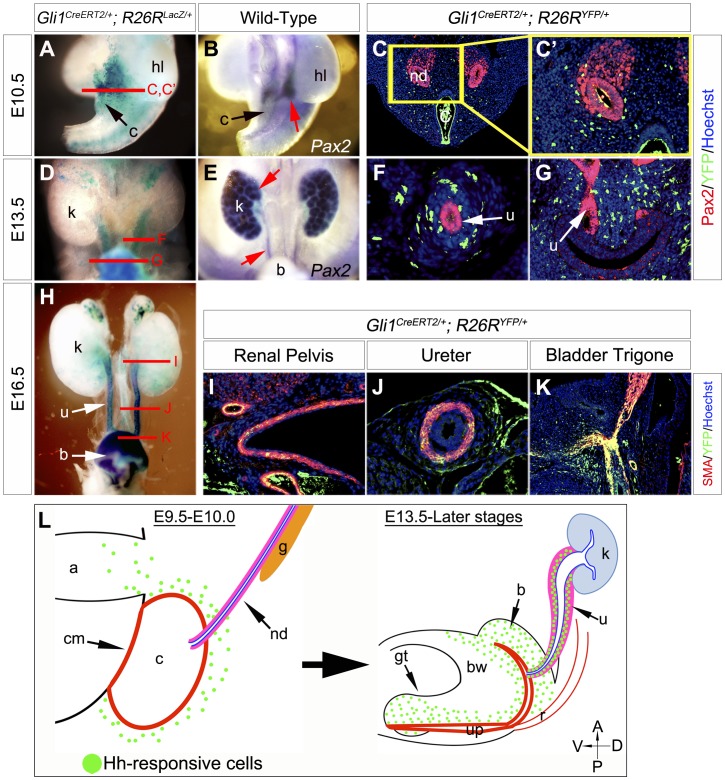
The contribution of Hh signal responsive cells to the upper urinary tracts. Hh signal responsive cell contribution assays were performed by utilizing the *Gli1^CreERT2^* system. Urinary tracts of embryos were analyzed at E10.5 (**A–C, C’**), E13.5 (**D–G**) or E16.5 (**H–K**) following TM treatment at E9.5. Gross morphology of *Gli1^CreERT2/+^; R26R^LacZ/+^* embryos with whole-mount X-gal staining (**A, D, H**). Red lines in **A**, **D** and **H** indicate corresponding areas for sections in **C, C’, F**, **G** and **I–K**. The expression of *Pax2* gene was analyzed at E10.5 and E13.5 to visualize the early urinary tract (**B, E**; red arrows). *Pax2* (red) and Hh-responsive cells (YFP: green) were co-stained in *Gli1^CreERT2/+^; R26R^YFP/+^* embryos (**C, C’, F, G**). A yellow box in **C** indicates magnified area in **C’**. At E13.5, urinary tissues were analyzed at the ureter (**F**) and bladder trigone (**G**) levels. Immunohistochemistry for the expression of SMA and YFP was performed in the transverse sections of the renal pelvis (**I**), ureter (**J**) and bladder trigone (**K**) region of *Gli1^CreERT2/+^; R26R^YFP/+^* embryos at E16.5. Summary of the Hh-responsive cell lineage analysis (**L**). The early Hh-responsive cells labeled by the *Gli1^CreERT2^* system are located in the peri-cloacal mesenchyme (**L**: left side). Part of such cells can contribute to the ureteral mesenchymal region in the late embryonic stage (**L**: right side). The *Gli1^CreERT2^*-labeled Hh-responsive cells are depicted as green dots. a: allantois, b: bladder, bw: body wall, c: cloaca, cm: cloacal membrane, g: gonad, gt: genital tubercle, hl: hindlimb, k: kidney, nd: nephric duct, r: rectum, u: ureter, up: urethral plate.

Our previous results suggested that the mesenchymal precursors for multiple urogenital structures, such as the bladder and external genitalia, are derived from the peri-cloacal mesenchyme (PCM) [29], [32]. The current study suggested that Shh signal at early embryonic stages is essential for the development of the upper urinary tract (Fig. 1). This suggests some of the Hh-responsive cells may contribute to the developing upper urinary tract. Examination of such a cell lineage is essential for understanding the pathogenic mechanisms of human congenital malformations. Therefore, genetic cell lineage analyses for Hh signal responsive cells were conducted by utilizing the *Gli1^CreERT2/+^; R26R^LacZ/+^* and *Gli1^CreERT2/+^; R26R^YFP/+^* systems. These strains are effective for analyzing the tissue lineage of Hh-responsive cells in many developmental contexts, such as the limb, central nervous and urogenital systems [29], [42], [62], [63]. Following TM treatment at E9.5, *Gli1^CreERT2/+^; R26R^LacZ/+^* mice were harvested and X-gal stained at E10.5, E13.5 and E16.5 (Fig. 2A,D,H). Most of the Hh-responsive cells in the urinary tracts were observed in the mesenchyme of the bladder, ureter and renal pelvis in such embryos. The ureteric bud began to form from the nephric duct at E10.5 (assessed by *Pax2* expression; Fig. 2B; red arrow). We also co-stained with YFP and Pax2 in *Gli1^CreERT2/+^; R26R^YFP/+^* mice (Fig. 2C,C’,F,G). A relatively small number of Hh-responsive cells (indicated by the YFP expression) were observed in the metanephric mesenchyme at E10.5 (Fig. 2C,C’). *Pax2* was expressed in the ureter epithelium and early kidney at E13.5 (Fig. 2E; red arrows). Hh-responsive cells are located in the mesenchyme around the ureter and the bladder at E13.5 (Fig. 2F,G). At E16.5, some of Hh-responsive cells also expressed alpha smooth muscle actin (SMA; Fig. 2I–K). The labeled cells were distributed broadly in the mesenchyme including SMA positive smooth muscle layers in the renal pelvis and the ureter (Fig. 2I,J). Such cells in the bladder were localized in the mesenchyme and smooth muscle layers (Fig. 2K). Of note, most of labeled cells were observed in smooth muscle layers in the bladder trigone region (Fig. 2K).

**Figure 3:**
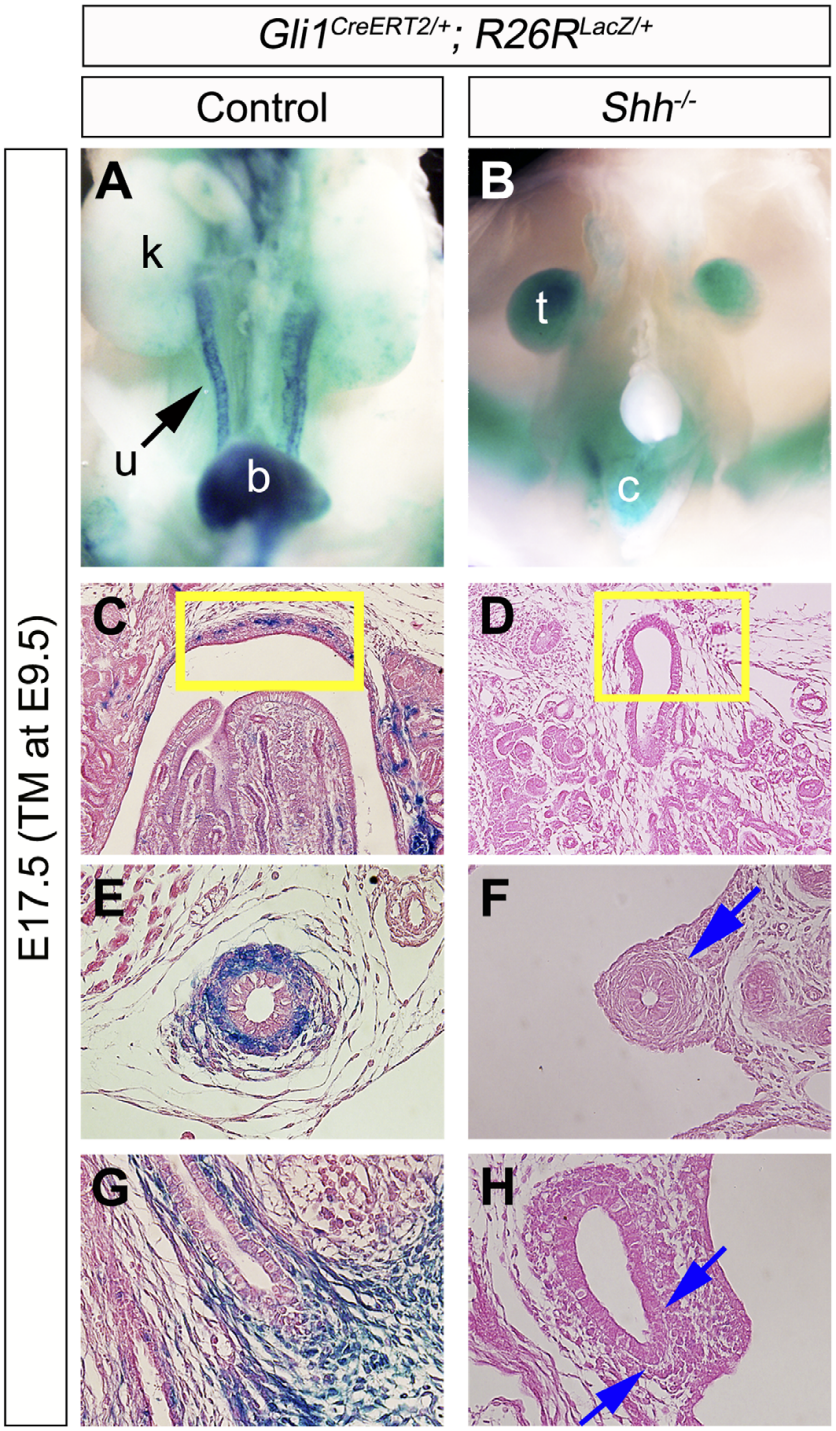
The contribution of Hh-responsive cells in *Shh* mutant urinary tracts. Hh-responsive cell contribution assay was performed by utilizing the *Gli1^CreERT2^; R26R^LacZ/+^* system. Urinary organs of embryos were analyzed at E17.5 following TM treatment at E9.5 (**A–H**). Gross morphology of X-gal stained embryos (**A, B**). A weak LacZ signal was detected in *Shh^−/−^; Gli1^CreERT2/+^; R26R^LacZ/+^*embryos in comparison to control embryos in the renal pelvis (yellow boxes in **C, D**), the ureter (**E, F**) and the bladder trigone region (**G, H**). Blue arrows indicate reduced LacZ signals in the urinary tracts of *Shh^−/−^; Gli1^CreERT2/+^; R26R^LacZ/+^* embryos. b: bladder, c: cloaca, k: kidney, t: testis, u: ureter.

The contribution of Hh-responsive cells was also examined in the *Shh^−/−^* background (Fig. 3A–H). We analyzed the population of LacZ positive cells in *Gli1^CreERT2/+^; R26R^LacZ/+^* and *Shh^−/−^; Gli1^CreERT2/+^; R26R^LacZ/+^* embryos at E17.5 after E9.5-TM treatment. Hh-responsive cells were not detected in the upper urinary tract of such embryos under the current experimental conditions (Fig. 3B,D,F,H). These results suggest that Shh signaling plays an essential role in the early Hh-responsive cells around the PCM region, which thus contribute to several upper urogenital tracts.

Moreover, we performed Hh-responsive cell contribution analyses at E18.5 embryos subsequent to TM administration at E11.5, E12.5 or E13.5. Few labeled cells were observed in the renal pelvis and the ureter region after TM treatment at E11.5 (Fig. S4A,D,G). In contrast to the administration of TM at E11.5, some LacZ positive cells contributed to the ureteral subluminal mesenchyme following the treatment at E12.5 or E13.5 (Fig. S4H,I). The embryos with TM treatment at E12.5 or E13.5 displayed weaker LacZ signals in the bladder mesenchyme than embryos with TM treatment at E11.5 (Fig. S4J–L). These results may be attributed to the difference of TM treatment timing and the tissues where Hh ligands emanated from. The Hh ligands are likely derived from the cloacal epithelia at the early embryonic stages. In fact, gene expression analyses revealed that *Shh* was not observed in the nephric duct at E10.5 (Fig. S1A,B). Additionally, Hh ligands are thought to be derived from the urothelia (bladder, ureter and renal pelvis epithelia) during the late embryonic stages (Fig. S1C–J).

### Constitutive Activation of Hh Signaling Induced Aberrant Smooth Muscle Development and Augmented Bmp Signaling

**Figure 4:**
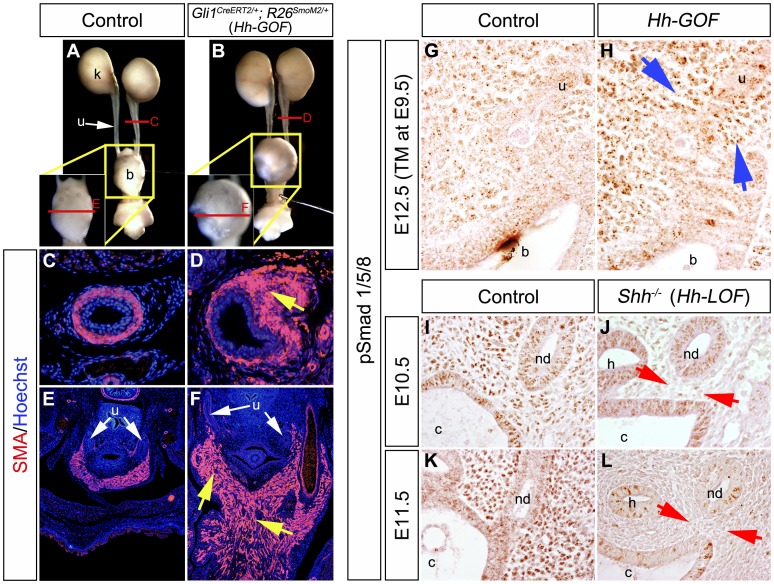
Constitutive activation of Hh signaling led to hyperplasia of urinary smooth muscles and augmented expression of phosphorylated-Smad1/5/8 in the urogenital mesenchyme. The gain-of-function experiments in Hh-responsive cells were performed by utilizing *Gli1^CreERT2^*; *R26^SmoM2^* (*Hh-GOF*) mice. Gross morphology of urinary tracts in control and mutant embryos at E17.5 after E9.5-TM treatment (**A, B**). Locations of the magnified bladder regions were indicated by yellow boxes in **A** and **B**. Levels of transverse sections in **C–F** are indicated by red lines in **A** and **B**. Immunohistochemistry for the expression of SMA (red) in transverse sections of the ureter (**C, D**) and the bladder trigone (**E, F**) regions. Yellow arrows indicate the abnormally expanded urinary smooth muscle layers. Immunohistochemistry for phosphorylated-Smad1/5/8 (pSmad) expression was also performed in *Hh-GOF* mutants with TM treatment at E9.5 (**G, H**). The *Hh-GOF* mutants displayed augmented pSmad expression in peri-cloacal mesenchyme at E12.5 (blue arrows). The *Shh^−/−^* (Hh signal loss-of-function mutants: *Hh-LOF* mutants) displayed a reduction of the pSmad expression at E10.5 and E11.5 (**I–L**: red arrows). b: bladder, c: cloaca, h: hindgut, nd: nephric duct, k: kidney, u: ureter.

*Shh* null mutants displayed urogenital abnormalities and a reduced expression of SMA (Fig. 1A–H). *Shh* conditional mutants also displayed urogenital abnormalities such as hydronephrosis, hydroureter and bladder hypoplasia by a temporal gene modulation (Fig. 1J–J”). To further examine the functions of Hh signaling during urogenital tract formation, we performed Hh signal gain-of-function (Hh-GOF) experiments. Mice carrying a constitutively activated form of Smoothened (*R26^SmoM2^*) and *Gli1^CreERT2^* alleles were utilized [40], [64]. The *Gli1^CreERT2/+^; R26^SmoM2/+^* (*Hh-GOF* mutants) displayed mesenchymal hyperplasia of the urinary tracts at E17.5 after E9.5-TM treatment (Fig. 4A–F). The ventral bladder wall was hyperplastic in comparison to the control and SMA expression domain was expanded in the mutant bladder wall (Fig. 4A,B,E,F). The width of the ureter smooth muscles was also expanded (Fig. 4C,D). The renal pelvis in *Hh-GOF* mutants did not display prominent phenotypes (data not shown). We then analyzed the activity of other growth factor signals in such mutants. The *Hh-GOF* mutants displayed augmented phosphorylated-Smad1/5/8 (pSmad) expression mainly in the *Gli1^CreERT2^*-activated mesenchyme at E12.5 (Fig. 4G,H; blue arrows). We also analyzed the expression of pSmad in *Shh^−/−^* embryos (Hh signal loss-of-function mutants: *Hh-LOF* mutants). Its expression was significantly decreased in the mutant PCM at E10.5 and E11.5 (Fig. 4I–L; red arrows). The pSmad expression in mutant epithelial layers was retained at E10.5, while it was reduced at E11.5 (Fig. 4J,L). Such significant differences of pSmad expression in the mesenchyme of both *Hh-GOF* and *Hh-LOF* mutants were also confirmed statistically (Figure S5). These results suggested that the activity of Bmp signaling may be regulated by the Hh signal during urinary tract formation.

### Temporal Inactivation of the *BmprIA* Gene in Hh-responsive Cells Resulted in Severe Hydroureter and Hydronephrosis Phenotypes; a Genetic Analysis of the Hh and Bmp Signaling Relay

**Figure 5:**
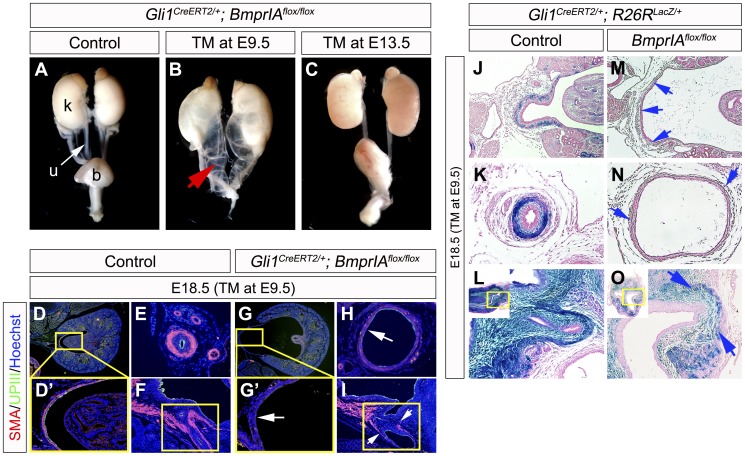
Conditional mutation of *BmprIA* gene resulted in urogenital organ defects and reduction in the number of Hh-responsive cells. *BmprIA* gene was mutated in Hh-responsive cells by utilizing *Gli1^CreERT2/+^; BmprIA^flox/flox^* mice. TM administration dependent hydronephrosis and hydroureter phenotypes (red arrow) were observed at E18.5 (**A–C**). The *Gli1^CreERT2/+^; BmprIA^flox/flox^* embryos displayed the reduced expression of SMA and UPIII in the renal pelvis (**D, D’, G, G’**), the ureter (**E, H**) and the bladder trigone region (**F, I**; yellow boxes). Locations of D’ and G’ were indicated in yellow boxes in D and G. White arrows indicate defective differentiation of cells based on marker expression. Hh-responsive cell contribution assays in *BmprIA^flox/flox^* conditional mutants were performed using the *Gli1^CreERT2/+^; R26R^LacZ/+^* system (**J–O**). The X-gal stained urinary organs from the control (**J–L**) and *BmprIA^flox/flox^; Gli1^CreERT2/+^; R26R^LacZ/+^* embryos (**M–O**) following the treatment with TM at E9.5. A reduced number of LacZ positive cells in the renal pelvis (**J, M**; blue arrows), the ureter (**K, N**; blue arrows) and the bladder trigone (**L, O**; blue arrows) were observed in *BmprIA^flox/flox^; Gli1^CreERT2/+^; R26R^LacZ/+^* embryos. b: bladder, k: kidney, u: ureter.

The current study suggested that Bmp signaling is linked with Hh signaling, based on the augmentation and the reduction of phosphorylated-Smad1/5/8 (pSmad) activities in *Hh-GOF* and *Hh-LOF* mutants (Fig. 4G–L). We next examined *Gli1^CreERT2/+^; BmprIA^flox/flox^* embryos to further investigate the possible relationship between Hh and Bmp signaling during upper urinary tract formation. We confirmed no significant alteration of *Gli1* expression in *Gli1^CreERT2/+^; BmprIA^flox/flox^* embryos at E12.5 with E9.5-TM treatment (Figure S6). Therefore, such allelic combination introduces the mutation of *BmprIA* in Hh-responsive cells and which is effective for analyzing the Hh-to-Bmp signaling relay in PCM cells. Embryos were treated once with TM at E9.5-E13.5 and the phenotypes were examined at E18.5. The *Gli1^CreERT2/+^; BmprIA^flox/flox^* embryos with TM treatment at E9.5 displayed severe phenotypes in the urinary tract such as hydroureter and hydronephrosis (Fig. 5B; red arrow). The mutant renal pelvis and ureter were widely dilated and the renal papilla was hypoplastic. These structures were surrounded by poorly differentiated smooth muscle cells (Fig. 5G,G’,H; white arrows). In addition, a reduction of UPIII expression was also observed in the renal pelvis region (Fig. 5D’,G’). The differentiation of urinary smooth muscle cells was also impaired in the bladder trigone of such mutants (Fig. 5F,I; white arrows). In contrast, mutants with TM treatment at E13.5 showed milder hydroureter-like phenotypes than E9.5-TM treated mutants (Fig. 5C). These results suggested that the Hh-to-Bmp signaling relay in the early PCM is essential for urinary tract development. Further experiments examined conditional inactivation of the *Bmp4* gene, which is a major ligand for BmprIA. However, *Gli1^CreERT2/+^; Bmp4^flox/flox^* embryos did not display prominent phenotypes under the current experimental conditions (data not shown).

Furthermore, the contribution of Hh-responsive cells was analyzed in *Gli1^CreERT2/+^; BmprIA^flox/flox^* embryos (Fig. 5J–O). The LacZ-labeled cells were observed in the mesenchyme of the renal pelvis, the ureter and the bladder trigone of control embryos following TM administration at E9.5 (Fig. 5J–L). On the other hand, few LacZ positive cells were observed in the renal pelvis and the ureter in *BmprIA* mutants (Fig. 5M,N; blue arrows). The LacZ positive cells were observed in the mesenchyme of the mutant bladder, but it was also reduced in number compared with the control embryos (Fig. 5L,O). These results suggested that Bmp signaling may therefore maintain the Hh-responsive cell population.

## Discussion

Recent advances of genetic techniques in animal models allow an analysis of the complex processes of embryonic development [65]–[67]. Genetic analyses of human patients have advanced greatly in recent years [68], [69]. Despite such advantages, the mechanisms for normal development and the pathogenic mechanisms for embryonic urinary systems remain obscure. Mesenchymal development in response to epithelially derived growth factors remains to be investigated. The current study showed the requirement of Hh signaling for the processes of urinary tract formation including its patterning and smooth muscle formation by analyzing a series of *Shh* mutant mice. Analysis of Hh-responsive cell contribution revealed that the progenitor cells for the mesenchyme of urinary organs are initially located in the peri-cloacal mesenchyme (PCM). A portion of those cells contribute to the urogenital mesenchyme and differentiate into urinary smooth muscles in response to Hh signaling. The conditional ablation of *BmprIA* gene in Hh signal responsive cell lineages suggested that the Hh-to-Bmp signaling relay in the PCM cells is essential for urogenital development. The current findings suggest new insights for the cellular origin and molecular requirements during urinary tract formation. The current results implicate the possible causative factors of urinary malformations such as CAKUT, one of the primary causes of kidney failure in infants.

### The Significance of Cloacal Hh Signaling during Urinary Tract Formation

Hedgehog signaling plays essential functions during urogenital development [26], [29], [30], [70]–[73]. *Shh* mutants displayed various abnormalities in the urinary tract such as the bladder agenesis, hydroureter and hydronephrosis [2], [6].

The current analysis of tamoxifen (TM) inducible conditional *Shh* mutants revealed the essential embryonic stages of *Shh* signaling for such urinary organ patterning and urinary smooth muscle differentiation. The time-controlled TM administration resulted in selective inactivation of the *Shh* gene in temporally CreER^T2^ activated tissues. Previous reports suggest the recombination event occurs within 6–12 hours and continues for up to 36 hours after TM administration [74], [75]. The *Shh* gene modulation in early embryonic stages (E9.5-E10.0) led to the urinary tract phenotypes such as hydroureter and hydronephrosis. In such stages, *Shh* gene is expressed in the cloacal epithelium and not in the epithelium of the nephric ducts. In addition, the ureteric bud is not yet formed in such stages. Accordingly, the *Shh* gene in the cloaca is selectively ablated in the urogenital tracts of such mutants. The current results also showed that Hh signal responsive cells in the peri-cloacal mesenchyme contribute to the urinary tract mesenchyme. These observations suggest that Hh signaling from the cloacal epithelia to the peri-cloacal mesenchyme plays an indispensable role for urinary tract development.

On the other hand, *Shh* was expressed broadly in the urothelia in later embryonic stages. Although the current *Shh^CreERT2/flox^* mice with TM treatment at later stages (e.g., E13.5) did not display prominent phenotypes, the *Hoxb7-Cre*; *Shh^flox/−^* mice, which are conditional mutants of the *Shh* gene in the mesonephric duct from E9.5, show delayed differentiation of ureteral smooth muscle, hydroureter and hydronephrosis phenotypes [2]. This suggests the necessity of early ureteral Hh signaling for proper urinary tract formation. These observations suggest cloaca derived and early ureter derived Hh signals would be critical for the Hh-signal-related urinary abnormalities.

In the current study, we showed that the reduction of Uroplakin3 (UPIII) expression in *Shh* conventional and conditional mutants. UPIII is a differentiation marker for urothelia and is known to be required for the water-impermeable properties of the urinary tract [52], [53]. Hence, the urothelial structures of these *Shh* mutants are suggested as rather immature. The reduction in the UPIII expression has also been reported to be observed in *Tbx18* mutants [56]. A reduction of the *Tbx18* expression was also demonstrated in *Shh* mutants. The reduction of the UPIII expression is likely caused by the reduction in the *Tbx18* expression. Both *Shh* and *Tbx18* are considered to function in the mesenchyme and the mutants for each gene display defects in the smooth muscle formation [2], [56]–[58]. The *Shh^CreERT2/flox^* embryos with E9.5-TM treatment still show *Gli1* expression in the ureteral mesenchyme, suggesting the presence of ureteral Shh signaling. Hence, the effect of such ureteral Shh seemed not affecting to the UPIII expression. From these observations, we speculate that the reduced UPIII expression may be attributed to indirect effects from the mesenchyme such as smooth muscles. Further analyses would illuminate the mechanisms for the urothelial maturation related with Hh signaling.

### Functions of the Hh-to-BMP Signaling Relay in Regulating the Urinary Tract Formation

The current results suggested that PCM cells can contribute to the urogenital mesenchyme and some of those cells differentiate into smooth muscle cells in later embryonic stages. We revealed that Hh signaling secreted from the cloacal epithelium is one of growth factors acting on PCM cells. In addition, we suggested its downstream signaling and assessed the function for urinary tract formation.

The current study found that the expression of phosphorylated-Smad1/5/8 (pSmad), an indicator of Bmp signaling activity, was decreased in *Shh* deficient (Hh signal loss-of-function: *Hh-LOF*) embryos. The reduced expression was mainly observed in the mesenchyme, but not in the epithelial layer at E10.5. In contrast, Hh signal gain-of-function mutants (*Hh-GOF*: *Gli1^CreERT2/+^; R26^SmoM2/+^*) showed the augmented pSmad signal mainly in the PCM at E12.5.

Regarding the correlation between Hh and Bmp signaling, several possibilities have been considered. Such possibilities could be (1) the regulation of the Bmp ligand expression by Hh signaling, (2) the regulation of the Bmp receptor expression by Hh signaling and (3) the modulation of the Bmp receptor activity and also the subsequent Bmp signaling by Hh signaling. Assuming that Hh signal activates the expression of Bmp ligands, the epithelial pSmad activity can also be augmented. However, an alteration of pSmad activity was mainly observed in the mesenchyme around the cloaca. In addition, we did not observe any significant phenotypes in the *Gli1^CreERT2/+^; Bmp4^floxflox^* embryos (data not shown). These observations may not support the possibility of number (1). However, it might be also possible that because of the mosaic induction of the *Gli1^CreERT2^* activity, only some PCM cells lost *Bmp4* expression. Hence, the effect of such *Bmp4*-deficient-cells may be masked by the Bmp4 proteins secreted from their neighboring cells. Further analysis is necessary to fully understand these phenomena. As for the possibility of number (2), we could not detect any alteration of the *BmprIA* expression in *Shh* mutants (data not shown). Accordingly, the current results suggest that Hh signaling likely functions as noted in number (3). Namely, it may modulate the mesenchymal Bmp signaling activity.

Several previous studies suggest an essential relationship between Hh and Bmp signaling [2], [27], [37]–[39]. However, the functions of Bmp signaling after receiving Hh signaling (Hh-to-Bmp signaling relay) remain obscure. To assess the significance of Hh-to-Bmp signaling relay, we analyzed *BmprIA* conditional mutant mice (*Gli1^CreERT2/+^; BmprIA^flox/flox^* mice). The *BmprIA* gene was modulated in Hh-responsive PCM cells by the administration of TM at E9.5. The *Gli1^CreERT2/+^; BmprIA^flox/flox^* mice displayed severe hydroureter, hydronephrosis and reduced smooth muscle differentiation in the urinary tract. The similarity of ureteral phenotypes between *Shh^−/−^* and *Gli1^CreERT2/+^; BmprIA^flox/flox^* mice also suggests that they are involved in the same signaling cascade in the peri-cloacal mesenchyme.

Several Bmp signaling genes are described as playing roles in the formation of the upper urogenital tract [76]–[79]. In fact, expressions of Bmp signaling genes (e.g., *BMP2*, *BMP7* and *BMPRIA*) are reported to be decreased in the fibrotic renal tissue of human hydronephrosis [80]. The *Bmp4* heterozygote mice display severe hydroureter and kidney hypoplasia with poorly differentiated ureteral smooth muscles [1], [81]. *Bmp4* may also have multiple biological functions such as prevention of apoptosis in the metanephric mesenchyme and serving as a chemoattractant for ureteral mesenchymal cells [33]. Such functions may be diverged spatiotemporally during urinary tract formation. The current analysis revealed a reduced number of the LacZ-positive cells in the *Gli1^CreERT2/+^; BmprIA^flox/flox^; R26R^LacZ/+^* mice in comparison to the control embryos. The mutation of the *BmprIA* gene in Hh-responsive mesenchyme resulted in severe hypoplasia of the mesenchymal region. These results imply a requirement of Bmp signaling for the maintenance of Hh-responsive PCM cell lineages.

In spite of the recent advances in the clinical care of the fetus, the mechanisms of urinary tract malformations such as CAKUT remain to be elucidated [18]–[20]. The current work revealed the possible influences of the cloaca, a transient embryonic cavity, to the pathogenesis of urinary tract abnormalities. The study revealed the unique contribution of the immature PCM to differentiated urinary tract mesenchyme and such contribution of PCM cells was regulated by the Hh and Bmp signaling receptions. These results may therefore help to elucidate the pathogenesis of urinary tract abnormalities.

## Supporting Information

Figure S1 **The expression of** ***Shh*** **and** ***Gli1*** **genes at whole-mount and sections of the urinary tract at E10.5 (A, B, K, L), E12.5 (C–F, M-P) and E13.5** (**G–J, Q–T**)**.** Red lines in **A, C, G, K, M** and **Q** indicate locations of the transverse sections in **B, D–F, H–J, L, N–P** and **R–T**. The *Shh* was expressed in the epithelia of the cloaca and the ureter (**A–J**). Red arrowheads indicate the expression of *Shh* in ureteral epithelia at E12.5 and E13.5. The *Gli1* was expressed in mesenchymal cells surrounding the cloaca at E10.5 (**L**). Its expression was observed in the urinary tract mesenchyme at E12.5 and E13.5 (**N–P, R–T**). b: bladder, c: cloaca, g: gonad, k: kidney, nd: nephric duct, u: ureter.
(TIF)

Figure S2 **The transverse sections of the urinary tract at E10.5, E11.5 and E12.5 (A–P).** Expression of *Pax2* in control (**A, C, E, G**) and *Shh^−/−^* (**B, D, F, H**) embryos. Expression of *Tbx18* in control (**I, K, M, O**) and *Shh^−/−^* (**J, L, N, P**) embryos. Red arrows indicate a reduced expression of the *Tbx18* gene around the nephric duct and the ureter. c: cloaca, nd: nephric duct, u: ureter, ub: ureteric bud.
(TIF)

Figure S3 **The expression of** ***Gli1*** **in the control (A, C, E) and** ***Shh^CreERT2/flox^*** **(B, D, F) embryos at E14.5 with E9.5-TM treatment.** Transverse sections of the bladder (**A, B**), bladder trigone (**C, D**) and ureter (**E, F**). Red arrowheads indicate the reduced *Gli1* expression.
(TIF)

Figure S4 **The contribution assays of Hh-responsive cells utilizing the** ***Gli1^CreERT2/+^; R26R^LacZ/+^*** **system.** Gross morphology and sections of X-gal stained urinary organs at E18.5 subsequent to TM treatment at E11.5 (**A, D, G, J**), E12.5 (**B, E, H, K**) and E13.5 (**C, F, I, L**). Yellow lines in **A–C** indicate the levels of the transverse sections in **D–L**. Transverse sections of the renal pelvis (**D–F**; yellow boxes), ureter (**G–I**) and bladder trigone (**J–L**; yellow boxes) regions. Blue arrows in **G–I** indicate weak activity of LacZ. b: bladder, k: kidney, u: ureter.
(TIF)

Figure S5 **Quantitative analysis on ratios of pSmad positive cells between wild-type and mutants.** The cell number of pSmad positive and negative cells in the defined three areas of 4 different sections was counted and ratios of pSmad positive cells were compared. Data were analyzed using the Student’s *t*-test or Welch’s *t-*test followed by the F-test. The ratio of pSmad positive cells was significantly reduced in *Shh^−/−^* embryos at E11.5 (**A**: Wild-Type: 0.718±0.05, n = 12, *Shh^−/−^*: 0.499±0.06, n = 12; *P*<0.001). The ratio of pSmad positive cells was significantly increased in *Gli1^CreERT2/+^; R26^SmoM2/+^* (*Hh-GOF*) mice at E12.5 (**B**: Wild-Type: 0.627±0.118, n = 12, *Hh-GOF*: 0.776±0.05, n = 12; *P*<0.05).
(TIF)

Figure S6 **The** ***Gli1*** **expression in the control and** ***Gli1^CreERT2/+^; BmprIA^flox/flox^*** **embryos at E12.5 with E9.5-TM treatment.** Its expression was not significantly altered in the mutant bladder trigone (**A, B**) and ureter (**C, D**) mesenchyme.
(TIF)
