# Supplementary figures and images for: The Hedgehog Signal Induced Modulation of Bone Morphogenetic Protein Signaling: An Essential Signaling Relay for Urinary Tract Morphogenesis
Source: PLoS One. 2012 Jul 30;7(7):e42245. doi: 10.1371/journal.pone.0042245 (PMC3408458; doi:10.1371/journal.pone.0042245)

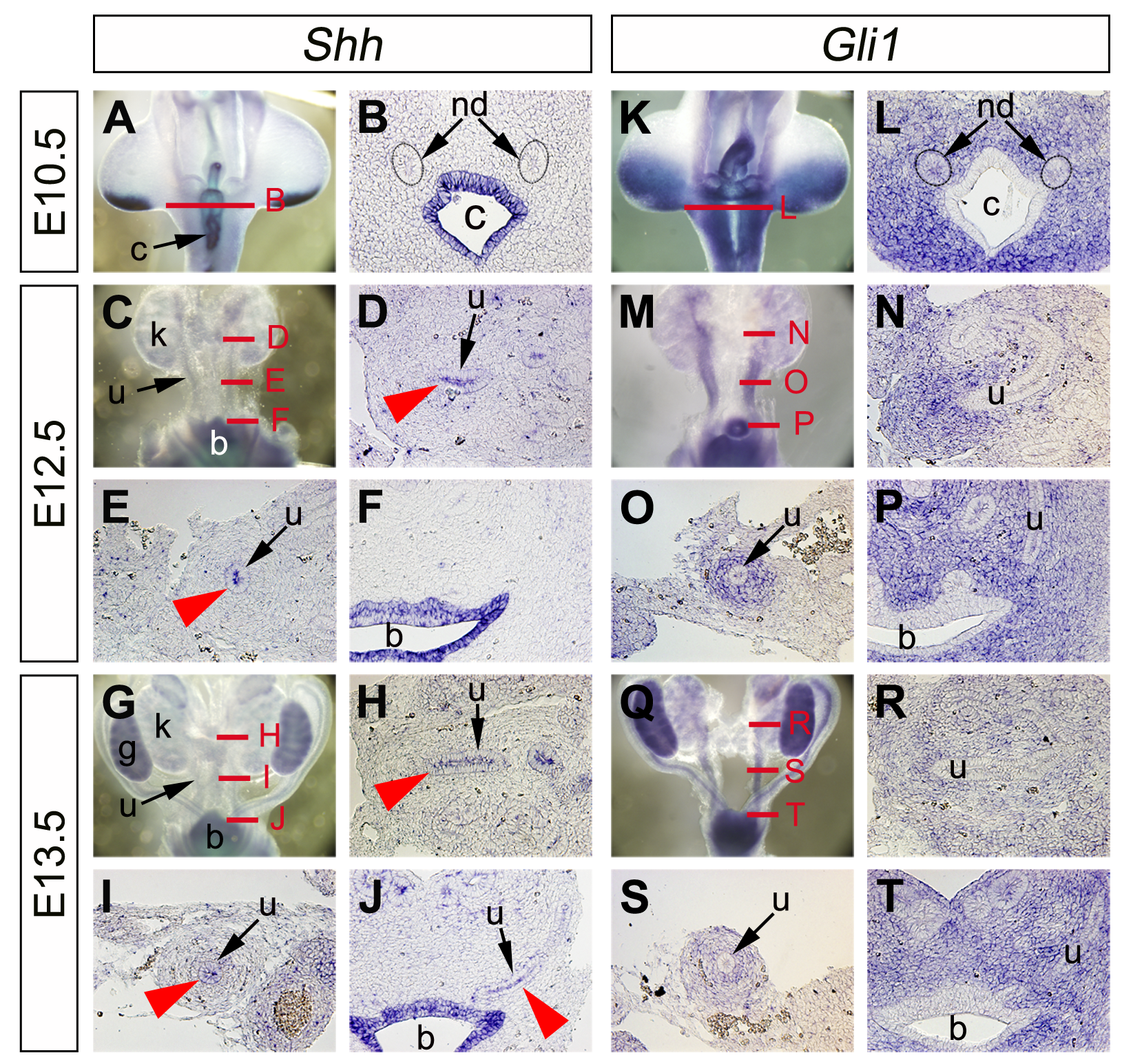

Supplement: Figure S1 — The expression of Shh and Gli1 genes at whole-mount and sections of the urinary tract at E10.5 (A, B, K, L), E12.5 (C–F, M-P) and E13.5 (G–J, Q–T). Red lines in A, C, G, K, M and Q indicate locations of the transverse sections in B, D–F, H–J, L, N–P and R–T. The Shh was expressed in the epithelia of the cloaca and the ureter (A–J). Red arrowheads indicate the expression of Shh in ureteral epithelia at E12.5 and E13.5. The Gli1 was expressed in mesenchymal cells surrounding the cloaca at E10.5 (L). Its expression was observed in the urinary tract mesenchyme at E12.5 and E13.5 (N–P, R–T). b: bladder, c: cloaca, g: gonad, k: kidney, nd: nephric duct, u: ureter. (TIF) [file pone.0042245.s001.tif]

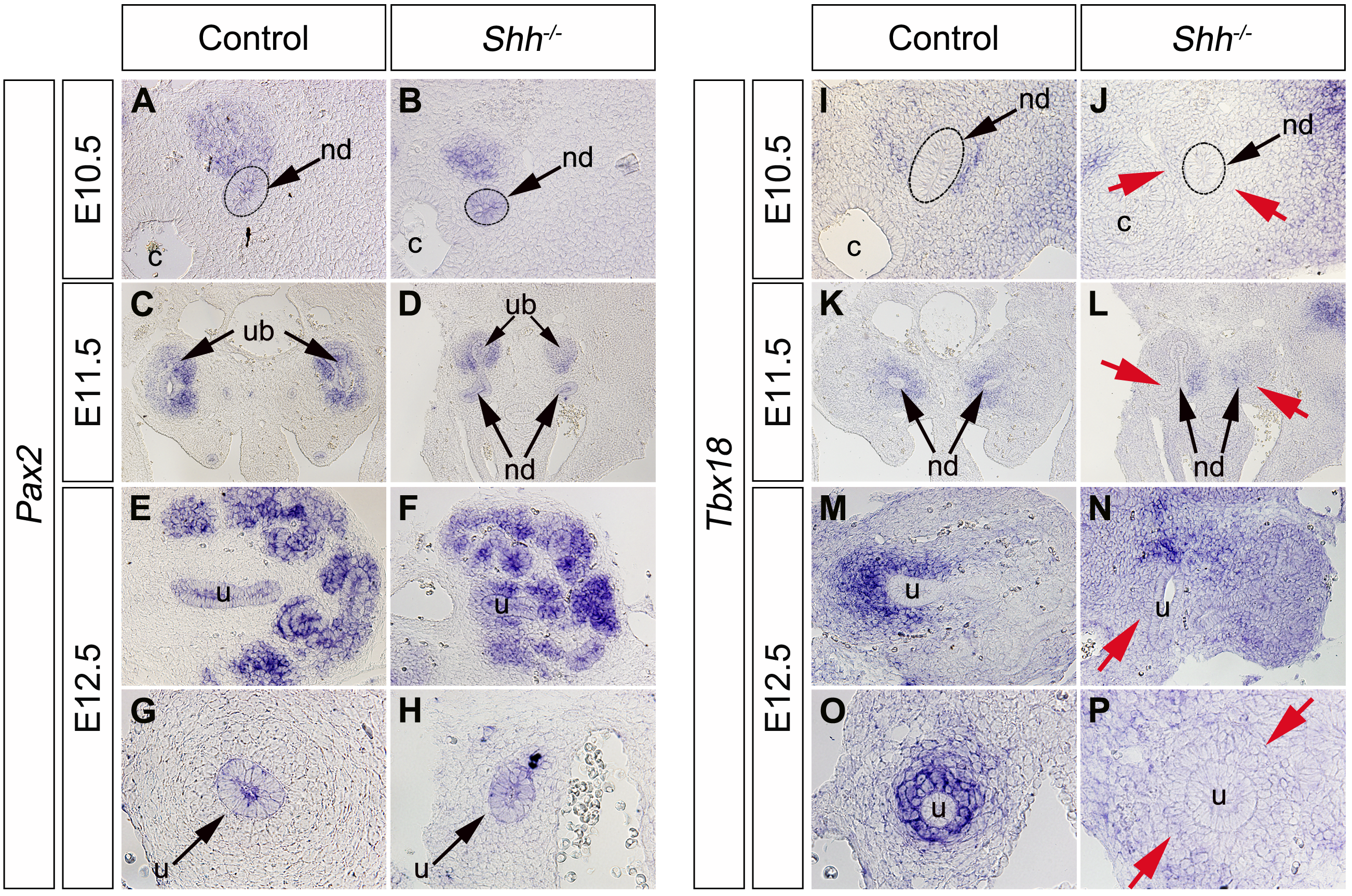

Supplement: Figure S2 — The transverse sections of the urinary tract at E10.5, E11.5 and E12.5 (A–P). Expression of Pax2 in control (A, C, E, G) and Shh−/− (B, D, F, H) embryos. Expression of Tbx18 in control (I, K, M, O) and Shh−/− (J, L, N, P) embryos. Red arrows indicate a reduced expression of the Tbx18 gene around the nephric duct and the ureter. c: cloaca, nd: nephric duct, u: ureter, ub: ureteric bud. (TIF) [file pone.0042245.s002.tif]

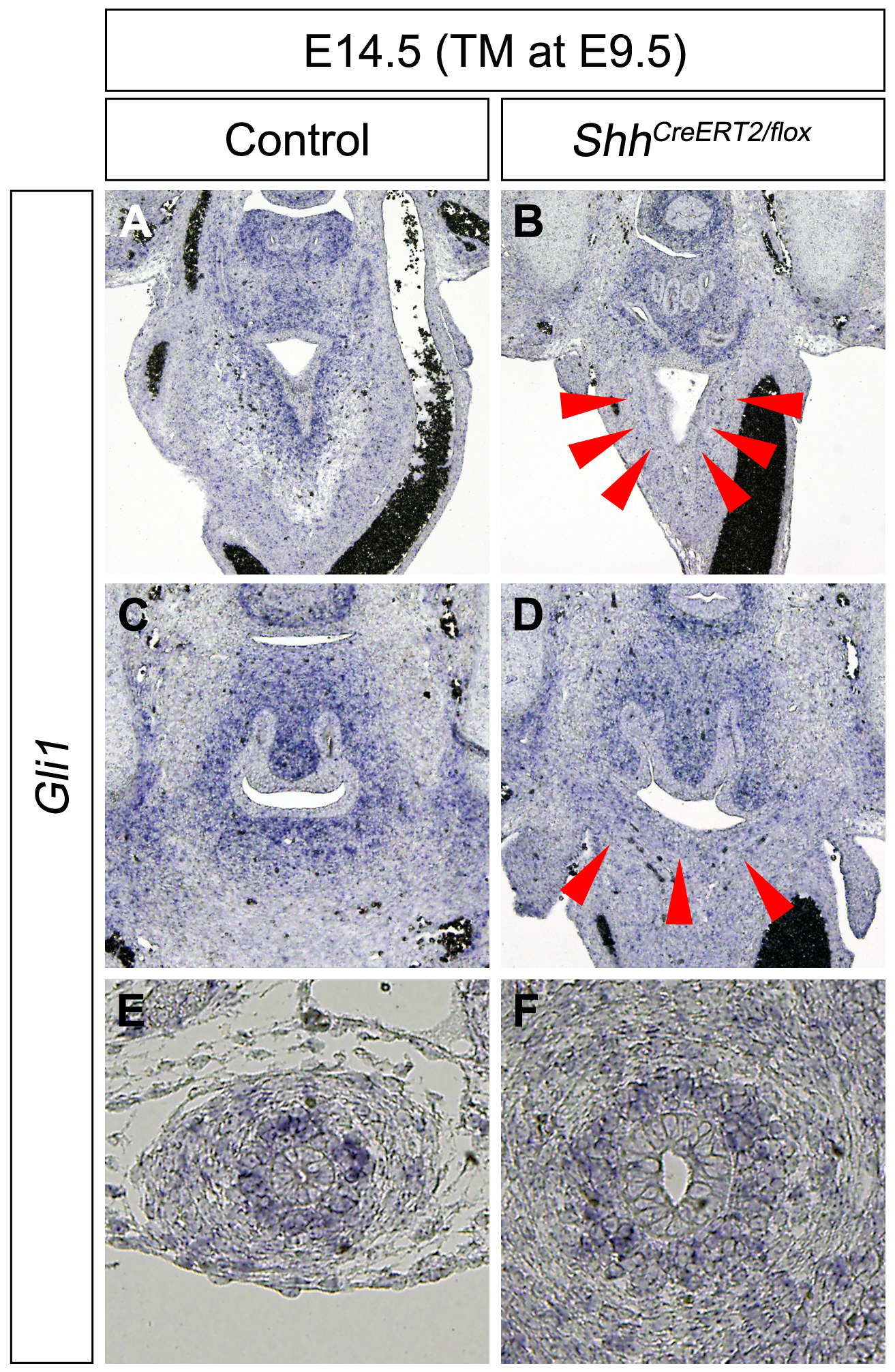

Supplement: Figure S3 — The expression of Gli1 in the control (A, C, E) and ShhCreERT2/flox (B, D, F) embryos at E14.5 with E9.5-TM treatment. Transverse sections of the bladder (A, B), bladder trigone (C, D) and ureter (E, F). Red arrowheads indicate the reduced Gli1 expression. (TIF) [file pone.0042245.s003.tif]

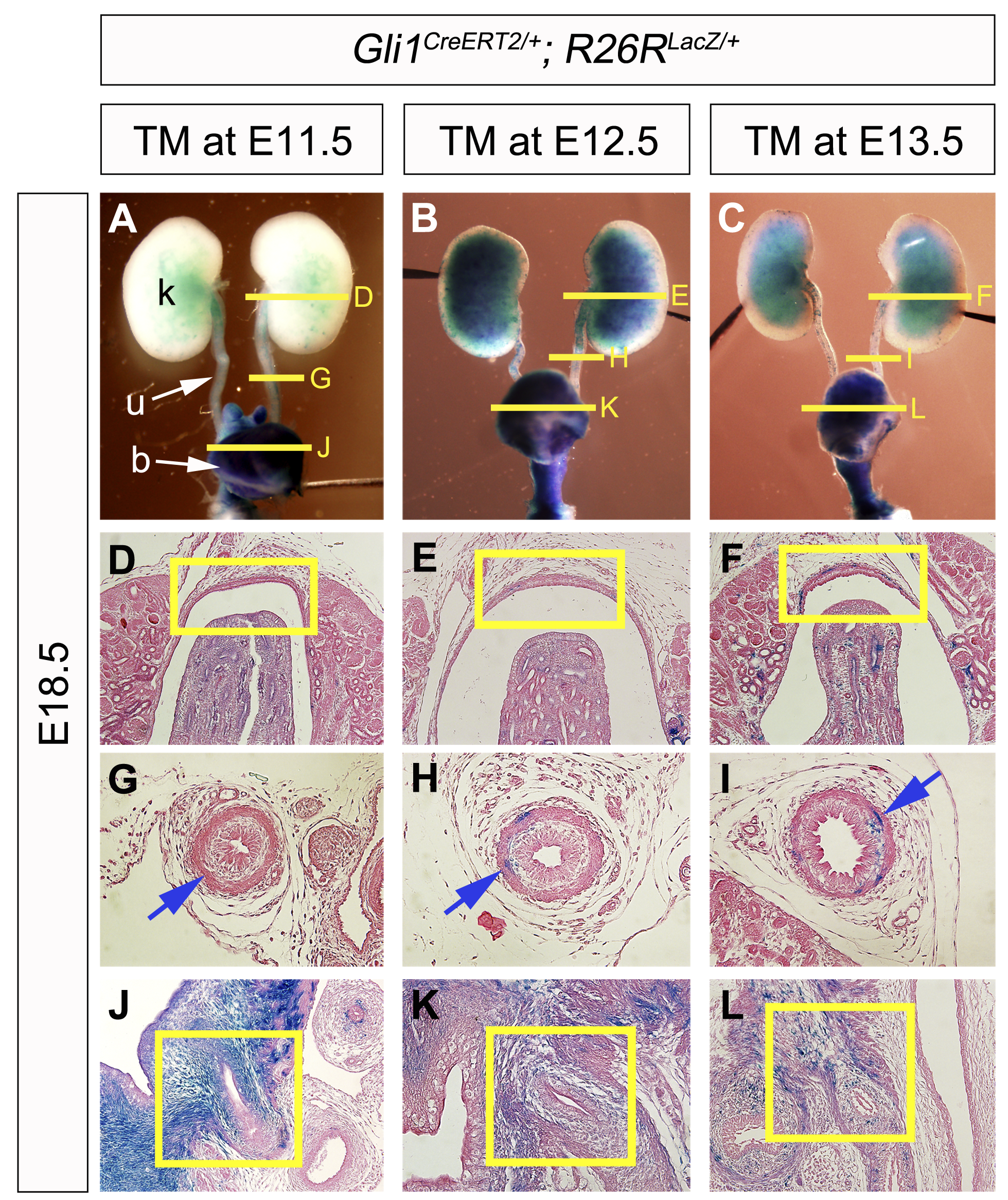

Supplement: Figure S4 — The contribution assays of Hh-responsive cells utilizing the Gli1CreERT2/+; R26RLacZ/+ system. Gross morphology and sections of X-gal stained urinary organs at E18.5 subsequent to TM treatment at E11.5 (A, D, G, J), E12.5 (B, E, H, K) and E13.5 (C, F, I, L). Yellow lines in A–C indicate the levels of the transverse sections in D–L. Transverse sections of the renal pelvis (D–F; yellow boxes), ureter (G–I) and bladder trigone (J–L; yellow boxes) regions. Blue arrows in G–I indicate weak activity of LacZ. b: bladder, k: kidney, u: ureter. (TIF) [file pone.0042245.s004.tif]

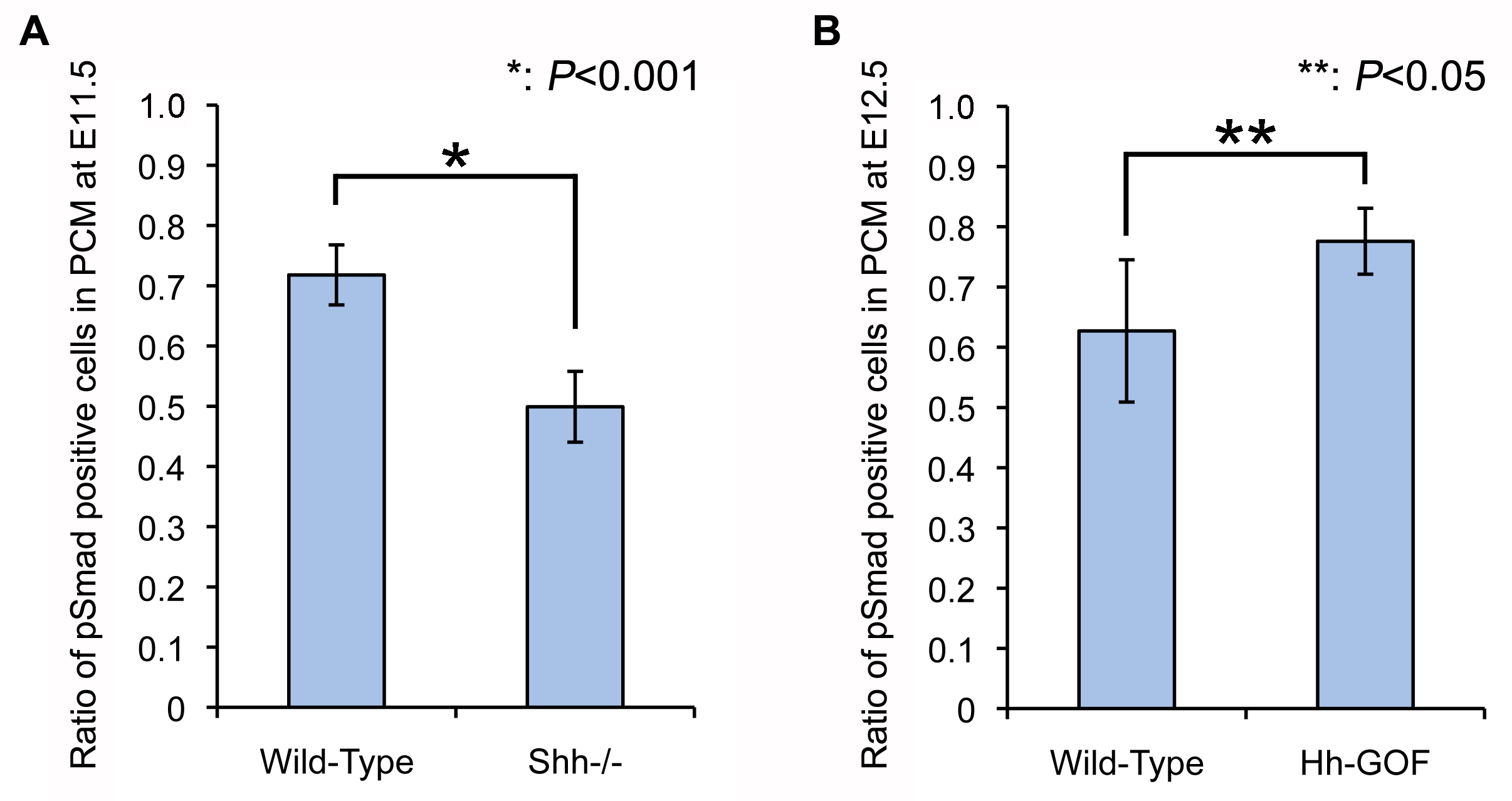

Supplement: Figure S5 — Quantitative analysis on ratios of pSmad positive cells between wild-type and mutants. The cell number of pSmad positive and negative cells in the defined three areas of 4 different sections was counted and ratios of pSmad positive cells were compared. Data were analyzed using the Student’s t-test or Welch’s t-test followed by the F-test. The ratio of pSmad positive cells was significantly reduced in Shh−/− embryos at E11.5 (A: Wild-Type: 0.718±0.05, n = 12, Shh−/−: 0.499±0.06, n = 12; P<0.001). The ratio of pSmad positive cells was significantly increased in Gli1CreERT2/+; R26SmoM2/+ (Hh-GOF) mice at E12.5 (B: Wild-Type: 0.627±0.118, n = 12, Hh-GOF: 0.776±0.05, n = 12; P<0.05). (TIF) [file pone.0042245.s005.tif]

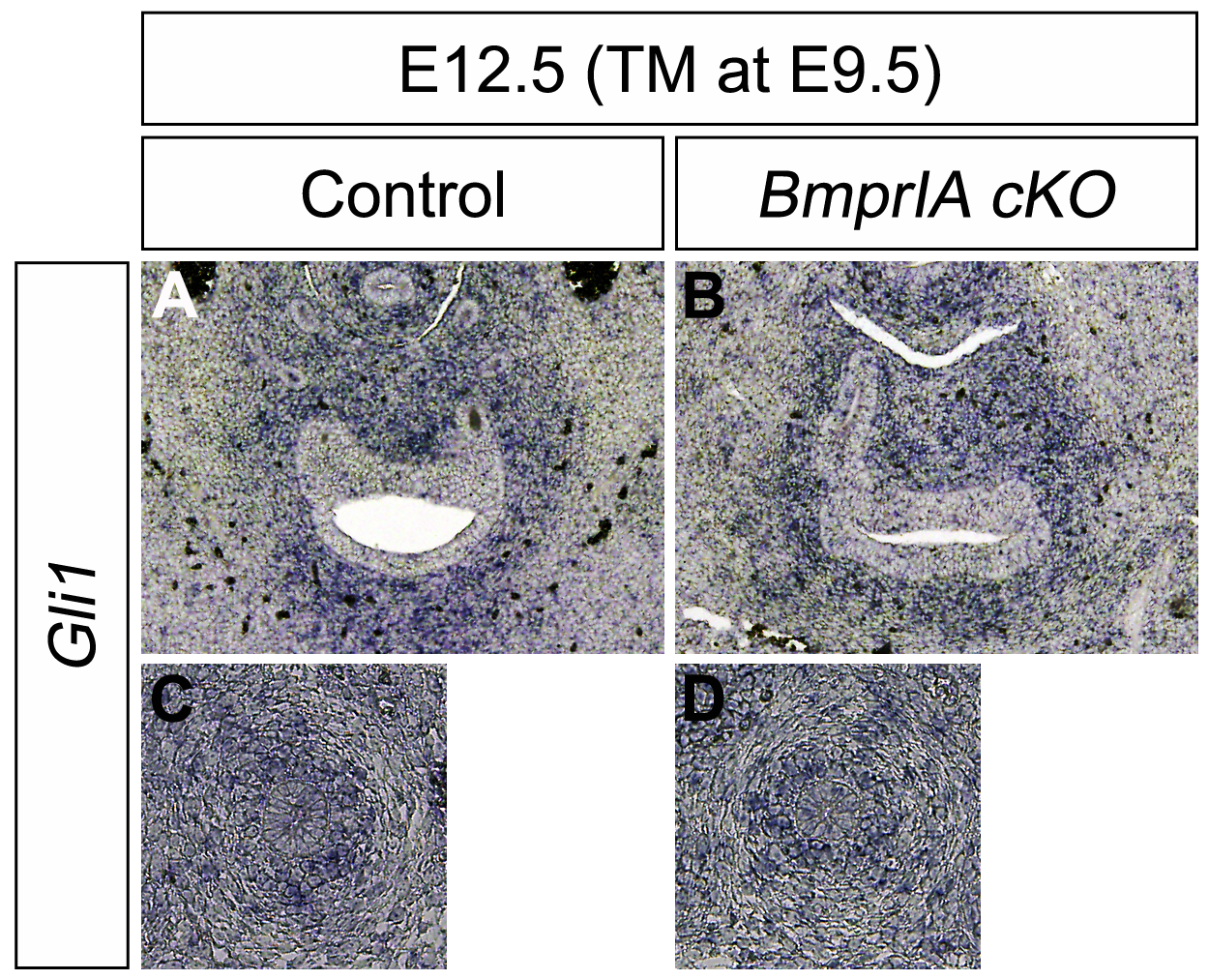

Supplement: Figure S6 — The Gli1 expression in the control and Gli1CreERT2/+; BmprIAflox/flox embryos at E12.5 with E9.5-TM treatment. Its expression was not significantly altered in the mutant bladder trigone (A, B) and ureter (C, D) mesenchyme. (TIF) [file pone.0042245.s006.tif]
